# Lessons Learned From Outbreaks of Shiga Toxin Producing *Escherichia coli*

**DOI:** 10.1007/s11908-012-0302-4

**Published:** 2012-12-05

**Authors:** Susanne Hauswaldt, Martin Nitschke, Friedhelm Sayk, Werner Solbach, Johannes K.-M. Knobloch

**Affiliations:** 1 Campus Lübeck, Department of Medical Microbiology and Hygiene, University Hospital of Schleswig-Holstein, Ratzeburger Allee 160, 23538 Lübeck, Germany; 2 Campus Lübeck, Department of Internal Medicine I, University Hospital of Schleswig-Holstein, Lübeck, Germany

**Keywords:** Shiga toxin producing *E*. *coli*, Enterohemorrhagic *E*. *coli*, Haemolytic uremic syndrome, O104:H4 outbreak, STEC decolonization

## Abstract

In 2011, a large outbreak caused by a Shiga toxin producing *E*. *coli* (STEC) occurred in Northern Germany, with a satellite outbreak in Western France, including the highest number of hemolytic uremic syndrome (HUS) cases ever encountered during a STEC outbreak. The outbreak strain was characterized as an enteroaggregative *E*. *coli* of serotype O104:H4 expressing a phage-encoded Shiga toxin 2. The majority of STEC infections and HUS cases were observed in adults, with a preponderance of the female gender. The outbreak imposed huge challenges on clinicians, microbiologists, and epidemiologists but also provided important new insight for the understanding of STEC infection. Thus, novel therapeutic strategies in the treatment of HUS in adults and for decolonization of long-term STEC carriers were evaluated. This review highlights the unusual features of the recent O104:H4 outbreak and focuses on emerging new strategies in diagnostics and treatment of acute STEC-related disease, as well as STEC long-term carriage.

## Introduction

Shiga toxin (Stx) producing *Escherichia coli* (STEC) were first described as Vero toxin producing *E*. *coli* (VTEC) leading to bloody diarrhea (hemorrhagic colitis) and the hemolytic uremic syndrome (HUS) about 30 years ago [1, 2]. HUS is characterized by hemolytic anemia, thrombocytopenia, acute kidney injury (AKI), and, in severe cases, neurologic complications. The natural reservoirs of STEC are ruminant animals, especially cattle. Transmission to humans usually occurs via contaminated food or water. Numerous outbreaks, as well as sporadic cases of STEC infections and HUS, have been documented worldwide. Obviously, the largest and best documented outbreaks were recorded in industrialized countries. This, however, does not necessarily mirror an increased frequency of STEC infections in developed countries but, rather, results from a reporting bias due to diagnostic resources, nationwide reporting systems, and publication activities [3]. Alternatively, in contrast to locally restricted farming in developing countries, modern industrialized large-scale food production might serve as a widespread vector in cases of food contamination.

STEC strains carry phages that encode Shigatoxins 1 and/or 2, also known as Vero toxins or Verocytotoxins [2, 4]. For both Stx 1 and Stx 2, several allelic variants are described [5]. Enterohemorrhagic *E*. *coli* (EHEC), the “classical” subset of STEC, possess the *eae* gene of enteropathogenic *E*. *coli* as an additional virulence factor conferring adherence to the intestinal mucosa. STEC not harboring *eae* were long believed to be less virulent but have also been shown to be the causative agent of STEC outbreaks [6, 7].

The pathogenesis of HUS development is incompletely understood [8]. In brief, STEC are incorporated by macrophages and M cells of the colon mucosa and produce toxin, which is released when the host cell undergoes apoptosis. After local and systemic distribution, Stx causes damage to vascular endothelial cells of the kidney and brain by direct cytotoxicity, as well as indirectly via cytokine secretion, complement activation, and subsequent generalized disease.

*E*. *coli* can be subclassified by O and H serotyping. STEC usually belong to a relatively limited number of O:H serotypes. O157:H7 is the serotype that was documented in the vast majority of HUS cases [9]. However, in some geographic regions, including Germany, non-O157 serotypes have been reported to account for up to half of HUS cases [10, 11].

In Germany, STEC were commonly recognized as pathogens causing rare but severe disease almost exclusively in younger children. Before 2011, about 1,000 infections per year and fewer than 100 cases of HUS were registered throughout Germany [12]. This review focuses on the 2011 outbreak of a STEC of serotype O104:H4, with unusual genetic properties leading to atypical epidemiologic and clinical presentation. Its impact on the scientific understanding of STEC is compared with historical data. New developments in the diagnosis and treatment of STEC and HUS are highlighted, as well as questions remaining open.

## The Recent German and Major Historical STEC Outbreaks

STEC primarily cause sporadic disease or small outbreaks limited to a few individuals. However, most information about STEC disease and its therapeutic implications was drawn from larger food-borne outbreaks, because the responsible strains were usually well characterized and epidemiological and clinical data were sufficiently available to gain statistically solid insight.

### Northern Germany Outbreak, 2011

The 2011 outbreak of a STEC O104:H4 in Northern Germany started at the beginning of May, reaching its peak on May 22. Until July 4, when the end of the outbreak was officially declared, 855 cases of HUS, 2,987 cases of acute gastroenteritis, and 53 deaths were registered in Germany [13, 14•]. Additionally, 83 EHEC cases and 54 cases of HUS were recorded outside Germany, most of them linked to travel to Northern Germany in May 2011 [14•]. Fenugreek sprouts were identified as the most likely vehicle of infection and were traced back to a single producer [15]. Despite all efforts, the strain could not be isolated from the implicated seeds. Shortly after the discovery of sprouts as the source of infection, a smaller outbreak by STEC O104:H4 was reported from Western France, including 24 patients with 7 cases progressing to HUS [16]. Here again, consumption of Fenugreek sprouts was associated with infections.

The outbreak proved a major challenge to clinicians, microbiologists, and epidemiologists, not only because of its severity, but also due to its unusual presentation. In contrast to earlier outbreaks, the majority of patients were adults, predominantly middle-aged and otherwise healthy women. The incidence rate of HUS (22 %) was higher than reported in previous outbreaks (1 %–15 %) [17••] and was accompanied by an unusually high number of cases with severe neurological symptoms (aphasia, seizures, and delirium) [18]. The incubation time was approximately 8 days [13], which is longer than the incubation period reported for STEC O157 during an outbreak in Washington in 1994 [19].

Early during the outbreak, the national HUS reference center identified the strain as being of serotype O104:H4 and as belonging to a clone (HUSEC041) originally isolated from an HUS patient in Germany in 2001 [20•]. Previously, there were reports of six sporadic cases of HUS caused by a STEC O104:H4 in Germany, France, Korea, and Georgia; however, molecular fingerprinting of the available strains revealed them to be not identical [21]. The 2011 outbreak strain combined virulence factors of both enteroaggregative *E*. *coli* (EAEC) and STEC, harboring the pAA plasmid of EAEC, as well as a Stx 2 producing phage, combined with an extended spectrum betalactamase encoding plasmid [20•], and was therefore called an “enteroaggregative STEC.” Whole genome sequencing was undertaken, and its results were also available early on during the outbreak [22–24]. Moreover, a multiplex polymerase chain reaction PCR assay for the parallel detection of several characteristic virulence factors (stx2, terD, rfbO104, fliC H4) was rapidly developed and made publicly available [20•].

Despite the observation that the constitutive Stx production of STEC O104:H4 was lower, as compared with “classical” O157:H7 strains [25], the proportion of patients developing HUS was greater than in most outbreaks before. This led to speculation about a possible hypervirulence of STEC O104:H4, potentially mediated by a facilitated Stx uptake due to the very efficient adherence of EAEC to the intestinal mucosa [26]. While the core genome of STEC O104:H4 is highly similar to other EAEC of the same serotype, the Stx-phage was shown to be most closely related to the phage of an O111:H- strain [25] and has probably been acquired quite recently in the phylogenetic history of the outbreak strain.

During the outbreak, the mean shedding period was reported to be more than 34 days for STEC O104:H4 in patients not receiving antibiotics [27••], with some of them still shedding the pathogen more than 1 year after the outbreak (unpublished data). These data indicate that the outbreak strain might be carried for longer periods, as compared with STEC O157, for which a maximal shedding period of 124 days was reported [28]. This difference might be explained by the enteroaggregative adherence to human cells.

### Historical Outbreaks With “Classical” STEC O157:H7

There are numerous reports on STEC O157:H7 as the most common serotype associated with HUS, especially in children. The historical outbreak most comparable to the recent German epidemic was likewise linked to sprouts and took place in Japan in 1996 (Sakai city outbreak). About 6,000 persons were affected after white radish sprouts had been served at school canteens, exposing 47,000 children to the contaminated food [29, 30]. As compared with the German outbreak in 2011, the incidence of HUS in the Sakai city outbreak was considerably lower (106 cases out of 6,000 infections). Also in 1996, another outbreak of a STEC O157:H7 in Scotland involved 512 persons, of whom 279 cases were microbiologically confirmed. In this outbreak, the majority of patients developing HUS were adults (28 of the 34). Contaminated meat products were identified as the outbreak source [31, 32]. Another massive outbreak of O157:H7 that gained particular attention happened in the western U.S. in 1993, comprising 501 cases, 151 hospitalizations, and 45 cases of HUS. This outbreak was linked to consumption of undercooked hamburger meat at a fast food chain [19] and promoted the (misleading) opinion that ground beef is the primary vehicle for STEC infections.

### Non-O157 Outbreaks

Several reports on non-O157 STEC underline their potential to cause sporadic disease as well as epidemics. One of the largest non-O157 STEC outbreaks was caused by STEC O111:NM in Oklahoma in 2008 [33]. Out of 341 cases with gastroenteritis, 71 patients required hospitalization. The HUS rate was 17 %, and more than 50 % of HUS patients were adults. In 1992, STEC O111:H2 was isolated from the stools of 5 out of 10 children hospitalized with HUS and of 3 healthy contact persons in South Picardy, France [34]. Later on, this strain was shown to adhere to epithelial cells in an aggregative pattern and to possess the EAEC virulence plasmid [35], while also producing Stx 2. However, this “intermediate” virulence strain affected only children, and the outbreak source remained unclear.

EAEC was consistently shown to have the potential of causing severe gastroenteritis in the absence of Shiga toxins. The largest EAEC outbreak occurred in Japan in 1993, when 2,967 children developed severe gastroenteritis after having consumed the same school lunch. Stools from 30 patients were studied, and 27 strains of an *E*. *coli* ONT:H10 were isolated. The strain adhered to epithelial cells in vitro in the typical aggregative pattern of EAEC but was negative for all virulence factors of diarrheagenic *E*. *coli*, including Shiga toxins except for the presence of the enteroaggregative heat-stable toxin.

These and other reports show the high diversity of STEC strains and the potential of pathogenic *E*. *coli* to acquire virulence factors by horizontal gene transfer, resulting in new hybrid phenotypes and “atypical” clinical presentations. This challenges the detection of new STEC strains and the prediction of their pathogenicity, underlining the need for further investigation.

## New Therapeutic Approaches During the STEC O104:H4 Outbreak

During the 2011 outbreak in Northern Germany, clinicians were confronted with a large number of mainly adult patients with HUS associated with severe hemolysis and neurological complications [18, 36]. A large proportion of patients required renal replacement therapy. However, we also observed some STEC-infected patients with severe neurological disorders but lacking renal or hematologic signs of HUS (unpublished data).

Prior to the outbreak, no standardized causative treatment existed for STEC-HUS, and randomized clinical trials approving any therapeutic concept to be beneficial beyond best supportive therapy were missing [37]. Therefore, different therapeutic concepts were rapidly proposed [38] on the basis of theoretical considerations and preceding observations, but without evidence for the effectiveness of such “best guess” strategies. Moreover, these ad hoc strategies were adjusted to new observations [39] made during the outbreak. Thus, individual medical centers used varying therapeutic regimens [17••], including plasmapheresis, glucocorticoids, and the anti-C5 monoclonal antibody eculizumab.

The use of plasmapheresis was based on observations from the Scotland outbreak in 1996, with 24 adult patients suffering from HUS [40]. In a multicenter case–control analysis of the 2011 German outbreak with STEC O104:H4, no short term benefit for plasmapheresis with or without additional application of glucocorticoids was found in 251 patients receiving plasmapheresis, as compared with 47 patients without plasma exchange [17••]. In an additional single-center analysis of 130 HUS patients, again no benefit of plasmaphesesis was observed [36], whereas the risk of severe AKI and neurological complication was increased [36]. Likewise, analysis of 90 children with HUS caused by STEC O104:H4 did not justify recommending plasma exchange in pediatric HUS [41]. This is in accordance with a recent prospective follow-up study of 274 children showing that plasma exchange therapy was associated with a poor long-term outcome for children with STEC-HUS [42]. Unfortunately, most of these studies are limited by their retrospective character and biased by indication, since sicker patients were more often treated with plasmapheresis. Taken together, the relevance for plasma exchange therapy in STEC-HUS is highly questionable and might even be adverse.

A small group of 12 patients with severe neurological complications were enrolled in a non-controlled trial with immunoadsorption therapy [43] after several other interventions were assumed to be ineffective. This included multiple plasmapheresis sessions and/or eculizumab application. Only 2 patients proceeded directly to immunoadsorption. Hence, the authors stated that antibodies could play a role in the pathogenesis of neurological disorders and that immunoadsorption might amend the neurological symptoms. This study is limited by very heterogeneous treatment procedures and a very small sample size.

At the end of May 2011, a case series of three children suffering from STEC-HUS was published reporting rapid clinical improvement under therapy with eculizumab [39]. Therefore, starting from the end of May, over 300 patients of the German outbreak were treated with eculizumab off-label, initially as a compassionate use trial that was transformed to a nonrandomized trial including 198 patients. Final evaluation of this trial is pending. A short-term outcome analysis of 67 patients treated with eculizumab outside the above-mentioned trial showed no significant therapeutic benefit, as compared with a matched control group not having received eculizumab [17••]. However, in many medical centers, eculizumab was administered only to patients who had no clinical improvement during plasmapheresis and/or were suffering from severe neurological complications, which might imply a selection bias. Therefore, no definite conclusions can be drawn concerning the effect of eculizumab on the course of HUS. However, the treatment with eculizumab can be considered as a save intervention, and therefore, its prognostic impact, as well as optimal time-point and dosage, should be evaluated in future prospective randomized trials.

A major issue discussed during the outbreak was the question of whether or not antibiotics should be used. In previous retrospective analyses of STEC outbreaks and sporadic infections, patients treated with antibiotics were found to have an increased risk of HUS development [32, 44]. This dogma was recently confirmed by a large multicenter trial investigating risk factors for the development of HUS in EHEC O157:H7 infected children [45]. Therefore, the use of antibiotics was strongly discouraged unless secondary complications made antibiotic treatment urgent. In our center, we strictly abstained from using antibiotics unless it was unavoidable. However, since the C5a antibody eculizumab disrupts the complement cascade and, thereby, increases the risk for bacterial meningitis [46], antibiotic meningitis prophylaxis was mandatory in patients receiving eculizumab. Because several in vitro studies did not show any induction but, rather, suppression of Stx expression by azithromycin, this antibiotic was selected for recommendation by the German Society of Nephrology in the ad hoc guidelines for meningitis prophylaxis to be given orally for 14 days [38]. The close monitoring of STEC-shedding in stool samples revealed that all patients receiving azithromycin were rapidly decolonized from the outbreak strain, while untreated patients remained positive for STEC significantly longer [27••]. In detail, among antibiotic-treated patients, long-term STEC carriage (>28 days) was observed in 1 of 22 patients (4.5 %; 95 % CI, 0 %–13.3 %), as compared with 35 of 43 patients (81.4 %; 95 % CI, 69.8 %–93.0 %) who were not treated with antibiotics (*p* < .001). All 22 patients receiving azithromycin had at least 3 STEC-negative stool specimens after the completion of treatment, and no recurrence of STEC was observed. Therefore, as a proof of principle, a 3-day course of oral azithromycin was offered as decolonization therapy to long-term carriers (>28 days) of STEC O104:H4 who were initially not treated with antibiotics, if they were restricted in their social or working life (e.g., ban from work). All 15 patients treated with azithromycin for STEC decolonization had negative stool specimens after the 3-day course, without any deterioration of renal function or development of other HUS-related symptoms [27••]. Therefore, successful decolonization treatment was extended to more than 40 patients, without any adverse effects until now (unpublished data). Such decolonization regimen has always to be weighed cautiously against the risk of other potential, pathogen-independent adverse drug side effects [47].

The in vitro finding that azithromycin does not induce Stx expression was confirmed recently for STEC O104:H4 by Bielaszewska et al. [48•]. Comparing the effect of subinhibitory concentrations of several antibiotics on the induction of Stx production of STEC O104:H4, they found that ciprofloxacin increased Stx production, while meropenem, rifaximin, tigecycline, and azithromycin did not [48•]. Interestingly, STEC O104:H4 appears to respond differently to antibiotics, as compared with the “classical” STEC strains O157:H7 [49]. In the situation of acute STEC disease, the differential response of various STEC strains to antibiotics requires an early evaluation of these interactions in order to enable precise warnings or recommendations concerning antibiotic treatment.

Despite the in vitro induction of Shiga toxin expression by quinolones, preemptive therapy of STEC-HUS patients with a combination therapy of meropenem and ciprofloxacin in one other medical center resulted in statistically significant reduction of death, seizures, and STEC shedding [17••].

However, it has to be taken into account that all promising results concerning the use of antibiotics for the treatment of STEC during the German STEC O104:H4 outbreak were retrieved either in patients suffering already from HUS or in clinically recovered, now asymptomatic long-term carriers with a shedding time of at least 28 days. Therefore, at present, no definite conclusions can be drawn for the use of antibiotics in acute STEC diarrhea. Future trials might further elucidate the pros and cons of this issue. Any antibiotics should be handled cautiously in patients with acute bloody diarrhea caused by STEC until their benefit is approved in controlled trials.

## Conclusion

During the Northern German outbreak of STEC O104:H4 in 2011, new aspects regarding the therapy of STEC infections and HUS were investigated retrospectively, raising new questions that still need to be answered for STEC disease in general. From a clinical point of view, novel treatment strategies like eculizumab need further prospective evaluation. The use of plasma exchange therapy in STEC-HUS can be regarded as obsolete. The previous dogma that antibiotics are absolutely contraindicated in STEC disease needs to be revised. Especially in patients with already established HUS, as well as in long-term STEC carriers who have recovered from their acute gastrointestinal manifestation, treatment with azithromycin has a beneficial effect on STEC decolonization. However, there is no approved indication for antibiotic treatment during the acute STEC-related bloody diarrhea. New diagnostic methods for immediate serotyping, as well as rapid analysis of phage induction and modulation of Shiga toxin expression by different antibiotics, are essential for clinical decision making (e.g., antibiotic therapy) in future STEC infections.
